# High throughput genome scale modeling predicts microbial vitamin requirements contribute to gut microbiome community structure

**DOI:** 10.1080/19490976.2022.2118831

**Published:** 2022-09-08

**Authors:** Juan P. Molina Ortiz, Mark Norman Read, Dale David McClure, Andrew Holmes, Fariba Dehghani, Erin Rose Shanahan

**Affiliations:** a School of Chemical and Biomolecular Engineering, the University of Sydney, Sydney, Australia; b Centre for Advanced Food Engineering, the University of Sydney, Sydney, Australia; c School of Computer Science, Faculty of Engineering, the University of Sydney, Sydney, Australia; d Charles Perkins Centre, the University of Sydney, Sydney, Australia; e Department of Chemical Engineering, College of Engineering, Design and Physical Sciences, Brunel University, London, UK; f School of Life and Environmental Sciences, Faculty of Science, the University of Sydney, Sydney, Australia

**Keywords:** Gut microbiome, genome scale modeling, computational biology, networks, interactions, cofactors, vitamins, enterotypes

## Abstract

Human gut microbiome structure and emergent metabolic outputs impact health outcomes. However, what drives such community characteristics remains underexplored. Here, we rely on high throughput genomic reconstruction modeling, to infer the metabolic attributes and nutritional requirements of 816 gut strains, via a framework termed GEMNAST. This has been performed in terms of a group of human vitamins to examine the role vitamin exchanges have at different levels of community organization. We find that only 91 strains can satisfy their vitamin requirements (prototrophs) while the rest show various degrees of auxotrophy/specialization, highlighting their dependence on external sources, such as other members of the microbial community. Further, 79% of the strains in our sample were mapped to 11 distinct vitamin requirement profiles with low phylogenetic consistency. Yet, we find that human gut microbial community enterotype indicators display marked metabolic differences. *Prevotella* strains display a metabolic profile that can be complemented by strains from other genera often associated with the *Prevotella* enterotype and agrarian diets, while *Bacteroides* strains occupy a prototrophic profile. Finally, we identify pre-defined interaction modules (IMs) of gut species from human and mice predicted to be driven by, or highly independent of vitamin exchanges. Our analysis provides mechanistic grounding to gut microbiome stability and to co-abundance-based observations, a fundamental step toward understanding emergent processes that influence health outcomes. Further, our work opens a path to future explorations in the field through applications of GEMNAST to additional nutritional dimensions.

## Background

The collective of self-organized microbes living in the human gut give rise to biological processes that modulate non-communicable disease (NCD) etiology.^1–5^ Gut strains adapt to the gut environment based on the metabolic attributes encoded in their genome. Molecules central to cell metabolism are considered essential nutrients when they must be provided by the environment. However, specific metabolic attributes allow strains the option of synthesizing such molecules when unavailable (optional nutrients). Such characteristics lead to the establishment of nutritional and bioenergetic exchanges between sets of strains with complementary metabolic attributes, resulting in the emergence of higher scales of community organization (higher order units). It is now understood that the microbial processes that influence human biology cannot be effectively explained by individual strains, but rather the resulting higher order units.^6–8^ These may take the form of specific co-abundance or interaction modules (IMs),^6–8^ or reflect broader aspects of microbial community assembly observed across human populations (such as community types or enterotypes)^9,10^ which likely represent differential nutritional and bioenergetic signatures.^11^ Although the mechanisms through which resources are exchanged have been described,^12,13^ and these can be readily related to genome evolution,^14^ the higher-order processes constraining emergent structures are yet to be defined, partially due to the inherent complexity within the community.

Metabolite-driven interactions in bacterial communities have been extensively documented and reviewed.^15–17^ Yet, microbial coenzymes constitute an underexplored aspect of the gut environment that has recently been gaining momentum, particularly those also categorized as human vitamins.^18–22^ Vitamins have well-characterized biosynthetic and acquisition pathways,^21^ and constitute essential^20,22^ and optional nutrients^23,24^ for gut commensals. Previous observations report vitamin-restricted diet/media had nearly no impact in a community of gut auxotrophic species relative abundance.^25^ Recent evidence suggest that colon-targeted vitamin supplementation can alter microbial alpha diversity.^26^ Further, vitamin auxotrophy has been identified as a driver of co-dependence and cooperation in a synthetic microbial community.^12^ Similar dynamics have been proposed around bacterial quinones, which encompass menaquinones (vitamin K2) and ubiquinones.^27,28^ Together, these characteristics strongly suggest that cofactor-driven microbial interactions are intrinsic to higher order units in the gut microbiome.

In addressing gut microbiome complexity, we adopt a constructive approach by focussing on the cofactor nutritional dimension and individual gut strains, to then infer how their metabolic attributes can influence higher order agent assembly. Targeting a group of well-characterized families of cofactors, we aimed to relate metabolic attributes (optional) and nutritional requirements (essential) of individual gut strains to gut community structure. Given the emerging interest and its structural relationship to menaquinones (vitamin K2), we include ubiquinone 8 (CoQ8) in the quinones family. Note that for simplicity we use the term vitamin to refer to every family of explored compounds even though some are not human vitamins (for example, CoQ8). We explore an assembly of 816 genome scale models (GSMs), part of AGORA (assembly of gut organisms through reconstruction analysis),^29^ which have been used to accurately predict metabolomics-based outcomes previously.^29–32^ GSMs are computational reconstructions of individual genomes that allow modeling of the metabolic attributes encoded^33^ while accounting for the metabolic network within the cell as a whole, where metabolic modules converge and communicate, influencing functional outcomes.^34,35^ This reveals what is truly metabolically feasible, as phenotype cannot be determined by presence/absence of specific genes or pathways alone.^22^ Modeling of GSMs allows fine tuning of growth media, inspection of fluxes and cellular inputs and outputs, and strain growth in terms of biomass generation enabling the assessment of the effect of the environment over functional properties. In doing so, GSM modeling can reveal what constitutes essential and optional nutrients for a strain under specific environmental conditions.

To comprehensively infer the metabolic traits of 816 AGORA strains we relied on our recently developed high throughput GSM modeling pipeline, GEMNAST (Genome Scale Model based Metabolic and Nutritional Assessment), based on COBRApy,^36^ capable of assessing GSM metabolism in a comprehensive range of nutritional environments. Based on our initial results, we explore how vitamin metabolic attributes and nutritional requirements of individual gut strains influence higher order unit structure, including those linked to human gut community assembly (enterotypes). This analysis represents a novel approach through which our ecological understanding of gut microbial community structures is enhanced.

## Results

### Exhaustive assessment of the vitamin auxotrophy spectrum among gut bacteria

Higher order units in the gut emerge from individual strains interacting with each other and their environment. To determine what drives higher order units within the vitamin nutritional dimension comprehensive characterization of the metabolic attributes and nutritional requirements of a representative sample of gut strains was required. Hence, we aimed to identify which of the eight vitamin families (vitamins from here onwards) in Table 1 constitutes essential (required), optional (synthesizable under the right environment) and non-nutrient (neither) vitamins for 816 AGORA strains. To do this, we modeled strain growth under a range of standardized environmental conditions derived from nutritionally rich, anaerobic, universally defined growth media (UDM, Methods) which contained the elements required for *de novo* biosynthesis of the vitamins in question.

**Table 1.** Biologically active vitamin forms and analogues that can be transported through the cell membrane and that play a role in AGORA GSMs’ metabolism were selected, then grouped by family for our analysis.^37^ Vitamin precursors that form part of UDM from which active forms can be synthesized are shown under the “Precursors in media” column. *In this study the abbreviation ‘K’ refers to menaquinones (vitamin K2) as well as the ubiquinone 8 (coenzyme Q), but excludes phylloquinone (vitamin K1).

Vitamins and analogues	Precursors in media	Family	Role in bacteria metabolism
Thiamine, thiamine monophosphate, thiamine pyrophosphate	Thiazole, cysteine, tyrosine, ribose	Thiamine (B1)	Tricarboxylic acid cycle dependent physiology
Riboflavin, reduced riboflavin, flavin adenine dinucleotide, flavin mononucleotide	Guanosine triphosphate, ribose	Riboflavin (B2)	Coenzymes associated with flavoproteins mainly involved in oxidative metabolism
Nicotinic acid, niacinamide, nicotinamide ribotide, nicotinamide adenine dinucleotide	Tryptophan	Niacin (B3)	Precursor for NAD⁺ and NADP⁺ (hydrogen transfer)
Pantothenic acid	Valine, alanine, methyl-oxovaleric acid, formaldehyde	Pantothenic acid (B5)	Precursor for Coenzyme A (acyl group carrier) involved in fatty acid metabolism and cell membrane integrity
Pyridoxine, pyridoxal, pyridoxamine, pyridoxal 5-phosphate	Glutamine, ribose	Pyridoxine (B6)	Coenzyme involved in transamination/deamination reactions
Folic acid, tetrahydrofolic acid, 5-methyltetrahydrofolate	pABA, glutamate	Folates (B9)	DNA replication and methylation (single-carbon metabolism)
Cobalamin I, cobalamin II, adenosylcobalamin	Methionine, cobalt, glycine, CoA	Cobalamins (B12)	Methionine and nucleotide synthesis
Menaquionine-7, menaquionine-8, demethylmenaquinone-8, ubiquinone-8	Chorismate	Quinones (K)*	Cell membrane electron flow and oxidative stress regulation

**Figure 1.**
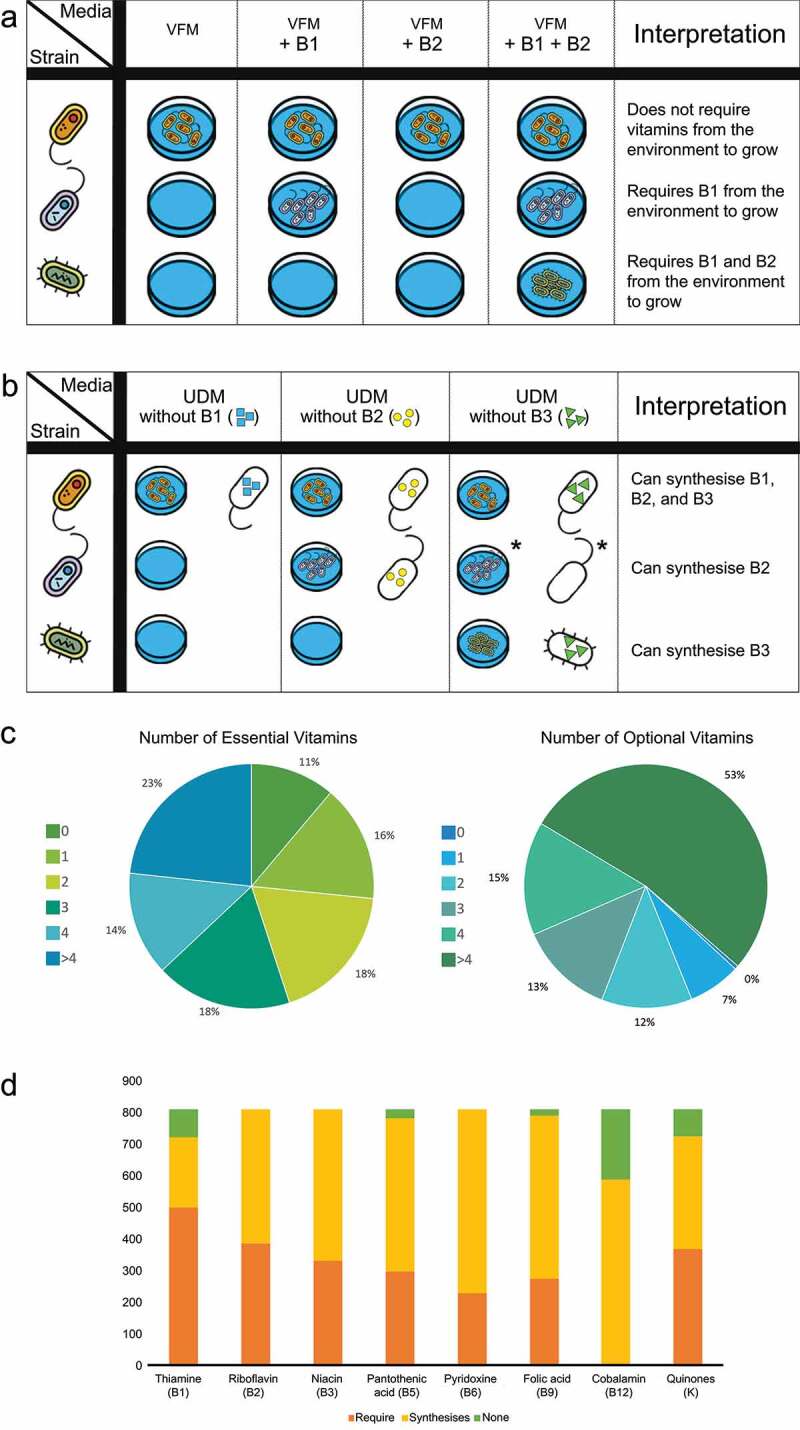
Graphic representation of our essential vitamins assessment (panel a) and optional vitamins assessment (panel b) experimental designs. Results of both assessments for 816 AGORA GSMs and eight vitamin families are summarized in panel d. Both utilize UDM (rich, anaerobic media), where all the 816 strains can grow as base media. a) Three hypothetical strains are modeled in four growth media formulations, the result of every combination of presence/absence of two vitamins (B1 and B2), which are added to vitamin-free UDM (VFM). Extrinsic vitamin requirements for the three strains are determined based on their individual growth profiles. b) The same three hypothetical strains are modeled in three growth media formulations, UDM missing a single vitamin (UDM – 1): B1, B2 or B3; if a strain is capable of growing in any of the given media its metabolic network is surveyed in search for the vitamin that was removed from UDM. If such family is identified, it is determined that the strain in question can synthesize it. *Strain 2 is capable of growing in UDM without B3 but does not synthesize B3 meaning that the vitamin is not required for strain 2 to grow and that strain 2 cannot synthesize it (non-nutrient). c) Pie charts with percentages of strains that presented a given number of essential and optional vitamins. d) Combined results from the analyses described in A and B. Vitamins were identified as either essential (“Require”), optional (“Synthesises”) or non-nutrients (“None”, when vitamins were not identified as essential nor optional) for the assessed AGORA strains.

To determine essential vitamins for 816 AGORA strains, GEMNAST recreated 256 *in silico* growth media formulations wherein vitamin-free universally defined medium (VFM) was supplemented with all combinations of presence/absence of the explored vitamins (Figure 1a). This ensured that the only limiting nutrients in the media were one or more of such families. Growth feasibility in a total of 208,896 *in silico* monocultures (256 per strain) was assessed by modeling strains’ whole metabolic network. As initial validation of our findings, we sought evidence of published *in vitro* vitamin requirements data. Soto-Martin *et al*. examined B vitamins requirements *in vitro* for eight of the strains in AGORA, among other.^22^ Based on the optical density (OD) readings provided by Soto-Martin *et al*. where strains achieved optimal or no growth in vitamin restricted media (Methods) we found that 79.2% of our predictions match *in vitro* outcomes (Additional file 1). Due to GEMNAST’s Boolean approach, eight *in vitro* cultures were considered inconclusive as sub-optimal growth of strains was reported (Methods).

Our essential vitamin assessment predicted vitamin dependence was widespread (Additional file 2). A majority of the strains (55%) presented more than two essential vitamins to grow above our predetermined threshold (0.09 gDW/h, Methods), while 126 strains (15.4% of our sample) had one essential vitamin. This demonstrates that even for a small set of vitamins, over 70% of strains in our dataset require at least two to be sourced from the environment (Figure 1c). B1 was identified as the most common essential vitamin as a total of 506 strains required it to be externally sourced (Figure 1d). The second and third most requisite vitamins were B2 and K, which were essential for 390 and 375 strains, respectively. In contrast, B12 was not essential in these growth conditions.

To complement our essential vitamins assessment, we aimed to identify optional vitamins for the strains in our sample. Identifying which AGORA strains are capable of synthesizing vitamins that are essential for others is necessary to reveal potential interactions in the gut where one strain sources the molecule to another. Further, complete biosynthetic pathways do not always determine phenotype; reconciling cellular-level metabolism and nutritional context is required. Hence, to further characterize AGORA strains’ vitamin metabolic attributes and properly discriminate optional vitamins from non-nutrients, GEMNAST modeled eight media formulations, each lacking a single vitamin family from our selection (Figure 1b). In total 6,528 *in silico* monocultures were performed. Here, we assessed a strain’s capability to grow *and* synthesize the vitamin missing from UDM (UDM – 1). Results revealed that vitamin biosynthesis is widely spread among the tested strains (Figure 1c, Additional file 2). We found that B12 is the most common optional vitamin in the tested growth conditions, with 600 strains being capable of synthesizing it while growing above our predetermined threshold (0.09 gDW/h); it was closely followed by B6 (optional for 583).

Coherently, we find that no vitamin was identified as both essential and optional for a given strain (Figure 1d). Combining our assessments of essential and optional vitamins notably reveals that B1, B5, B9, B12 and/or K are non-nutrients for a portion of strains in our sample (Figure 1d). Thus, biosynthetic pathway presence/absence is not sufficient to independently predict a vitamin metabolic attributes. The studied strains presented on average three essential and four optional vitamins for the tested growth conditions (Additional file 2).

To better understand the mechanisms mediating vitamin interactions we explored vitamin active export capabilities with GEMNAST. We found that, besides niacin, which was actively exported by 430 strains, export of synthesized (optional) vitamins is a rare occurrence within strains in our dataset (Additional file 3). This may imply that these molecules are not actively made available to other gut microbes, and are mainly passively exchanged; for example, vitamin transfer after cell lysis.^13,38^ However, it is also plausible that specific mechanisms for vitamin cross-feeding are yet to be described and therefore not represented within the AGORA framework. Overall, our findings suggest that gut strains present broadly complementary vitamin metabolic attributes and that passive/active inter-strain vitamin exchanges can occur in the gut microbiome, which can enable the emergence of higher order units.

### The microbial vitamin auxotrophy spectrum is occupied by discrete vitamin capability groupings

Interpreting microbiomes in functional terms can greatly aid in understanding the gut ecosystem. Across the surveyed strains, we identified 84 different essential vitamin profiles, plus a group of 91 strains with no essential vitamins in the tested nutritional conditions (prototrophs). Meanwhile, 138 optional vitamin profiles were identified among the 816 strains assessed. Such findings revealed significant overlap among strains’ vitamin metabolic attributes (functional redundancy). Hence, we sought to explore the distribution of strains in the auxotrophy spectrum aiming to identify broad vitamin capability groups that could improve our ability to parse vitamin-based functional complementarity and community structure. Accordingly, we performed hierarchical agglomerative clustering followed by non-linear two-dimensional clustering (Additional file 4), which revealed discrete groupings in our dataset.

**Figure 2.**
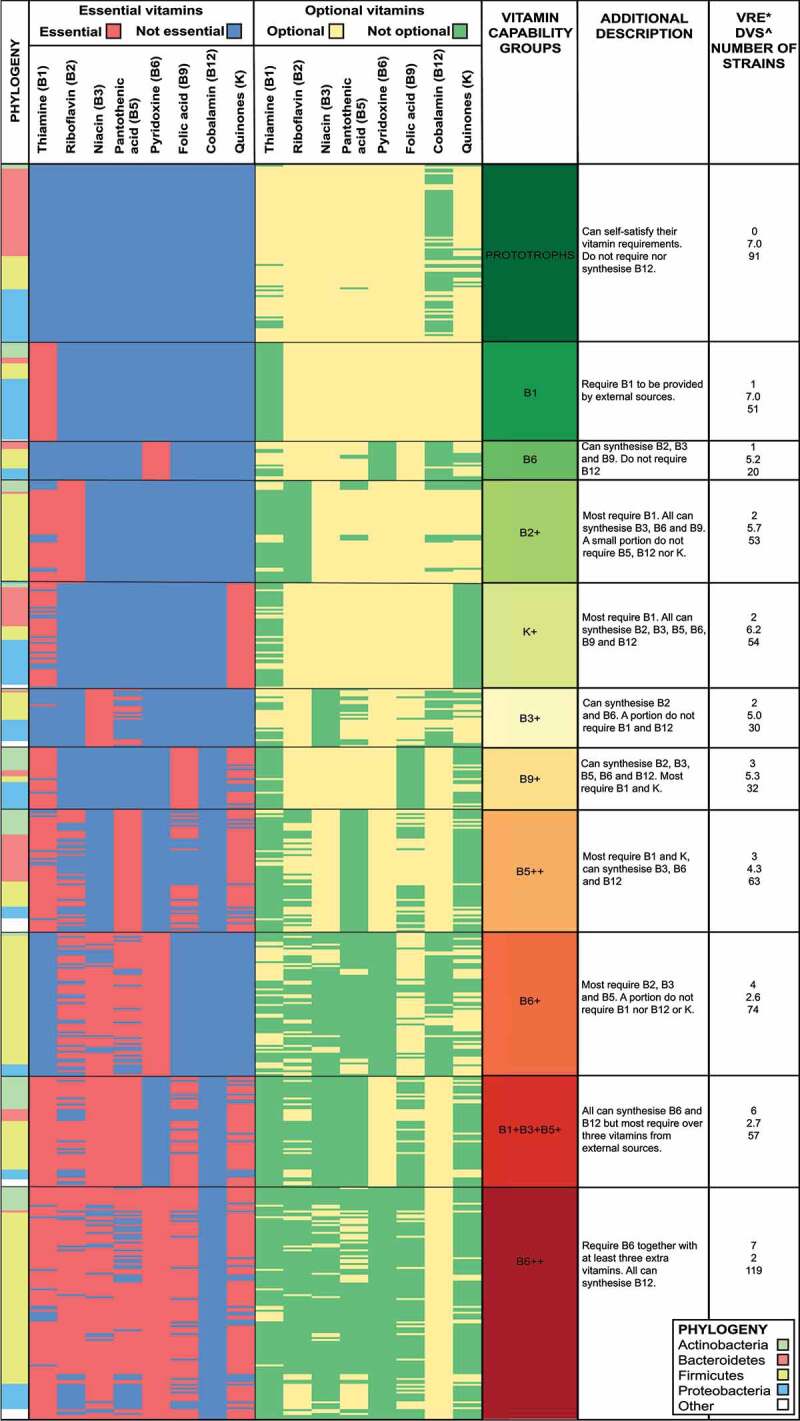
Essential and optional vitamins of the 644 strains mapped to a vitamin capability group. Each row represents a unique strain. Strain names are omitted for readability purposes (Refer to Additional file 2 for strain-specific details). The first column shows strain phyla by color. The second column details vitamins that are required from the environment (essential). The third column shows vitamins that strains can synthesize (optional). Contrast between both columns reveals groups of strains that do not require, nor synthesize specific vitamins (non-nutrient). Strains are grouped by “Vitamin Capability Groups”, based on essential vitamin profiles. Capability groups are named based on common requirements: A plus (+) sign next to a functional group name indicates strains in that vitamin capability group require one to three extra vitamins (other than the one(s) already indicated in the name); a double plus (++) indicates strains in the group require more than three extra vitamins from the environment. Colors assigned to vitamin capability groups range from dark green to dark red based on their location in the prototrophic to auxotrophy spectrum, respectively. Additional characteristics of each group are also provided (“Additional Information”). Right-most column displays 1) *Number of observed vitamins required from the environment (mode) 2) ^Detected vitamins synthesized (average) 3) Number of strains by vitamin capability group.

Informed by the above mentioned clustering approaches and following careful manual inspection, final capability groups were devised. In total, 79% (644 out of 816) of the stains in our sample were mapped to one of eleven vitamin capability groups, which were distributed along the auxotrophy to prototrophy spectrum (Figure 2). The additional 21% of strains in our sample presented vitamin requirements that were incompatible with capability groups patterns. Notably, we find that strains from the *Bacteroidetes* phylum were largely mapped to more prototrophic capability groups while highly auxotrophic capability groups are characterized by strictly anaerobic members of the *Firmicutes* phylum (Figure 2, Additional file 2). Figure 2 shows that vitamin capability groups, based on essential vitamin profiles, also present similar optional vitamin profiles. This suggests that such strains likely occupy similar niches within gut communities where evolutionary pressures led to the loss, or conservation, of biosynthetic pathways to guarantee survival/improve fitness.^39^ Based on group-level vitamin metabolic attributes, the Prototrophs group is the only one where strains can self-satisfy all their vitamin requirements and can further serve as a B2, B3, B5, B6, and/or B9 source for more environmentally dependent clusters (Figure 2). Meanwhile, other groups require external vitamin sources. For example, strains in group B2+ require B2, and most require B1, to be sourced by the environment. However, such strains are well positioned to source those from other groups with B3, B6, and/or B9 (Figure 2). Thus, broad complementary profiles can be observed at the vitamin capability group-level.

### Vitamin capability groups reveal marked differences between enterotype indicative taxa

**Figure 3.**
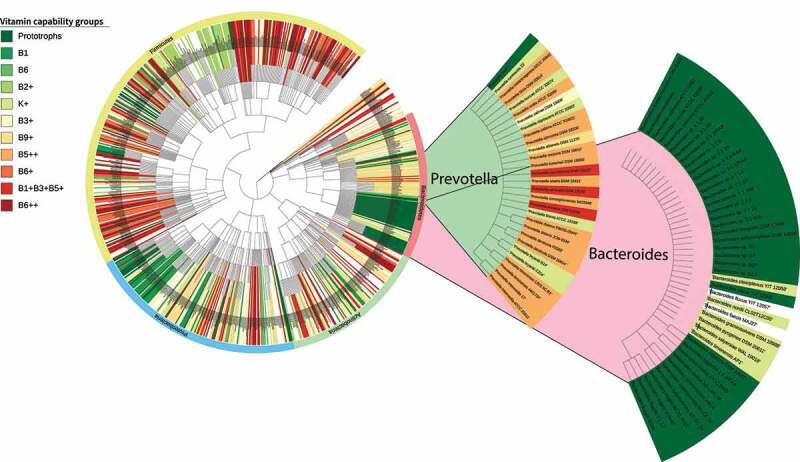
Phylogenetic tree of AGORA gut strains.^29,40^ Strain names are highlighted to clade level with the color that corresponds to the vitamin capability group they were mapped to; strains in white were not assigned to any vitamin capability groups. Colors assigned to vitamin capability groups range from dark green to dark red based on their location in the prototrophic to auxotrophy spectrum, respectively. Two independent trees including strains from the Bacteroides and Prevotella genera from our analysis show an important difference in terms of vitamin capability group membership.

To identify if previously observed patterns of community structure (such as enterotypes) are related to our functional categorization (vitamin capability groups), we aimed to assess the extent to which vitamin metabolic attributes are evolutionarily conserved. A phylogenetic tree, including 806 strains from our sample, was built using the National Center for Biotechnology Information’s (NCBI) Taxonomy Browser^40^ (Figure 3). Strains mapped to vitamin capability groups were identified within this tree revealing that while there is an observable consistency at the clade level, there is not a consistent evolutionary level of conservation of vitamin metabolic attributes. For example, the Prototrophs functional group was composed of strains from various genera, including *Streptococcus, Bacillus, Bacteroides, Escherichia*, and *Pseudomonas*. On the other hand, strains within the same genus were often split into different capability groups: for example, members of the *Blautia* genus were mapped to the Prototrophs (*Blautia hansenii* DSM 20583), B1 (*Blautia obeum* ATCC 29174), and B6+ (*Blautia hydrogenotrophica* DSM 10507) capability groups. Interestingly, strains from the *Prevotella* and *Bacteroides* genera, which are often identified as keystone taxa defining differential microbial community assemblages or “enterotypes” in the human gut,^10^ were largely mapped to different vitamin capability groups. Out of the 44 *Bacteroides* strains in our analysis, 36 were identified as prototrophs, while five were mapped to K+, a highly prototrophic group. Meanwhile, *Prevotella* strains were mostly mapped to the B5+ group (17 out of 29) while the rest were spread among five other vitamin capability groups (Figure 3), most of which displayed a high number of essential and a low number of optional vitamins.

Our mapping of vitamin capability groups onto enterotype indicators raises the possibility that these may reflect processes that drive community structure. The marked contrast between the vitamin capability groups to which enterotype dominant taxa were mapped led us to further explore the metabolic attributes of *Prevotella* strains, aiming to pinpoint how such attributes may impact community structure. Inspection of our vitamin metabolic attributes data (Additional file 2) showed that members of *Streptococcus, Enterococcus*, and *Lachnospiraceae*, which have been associated with the *Prevotella* enterotype in a prominent study,^10^ present metabolic profiles that complement those of the assessed *Prevotella* strains. Members of the *Lactobacillaceae* and *Ruminoccoccaceae* families were also found to be suitable complements to *Prevotella* vitamin metabolic attributes. For instance, *Prevotella brevis* ATCC 19188 can synthesize B2, B3, B5, B6, and B9 but requires a source of B1 and K, which can be provided by *Lactobacillus pentosus* KCA1 or *Lactobacillus johnsonii* DPC 6026, while both *Lactobacillus* strains require the vitamins *Prevotella brevis* ATCC 19188 can synthesize. Interestingly, there was no complementarity between *Prevotella* strains and members of the *Bacteroides, Anerostipes, Parabacteroides, Alistipes* genera or the *Enterobacteriaceae* family, all of which have been associated with the *Bacteroides* enterotype.^10,41,42^

### Vitamin dependent/independent IMs emerge from member strains’ metabolic attributes

Intermediate degrees of organization, what we refer to as IMs, can occur between the cellular and whole-community levels, and these have also been associated with health outcomes.^6–8^ Hence, relying on the vitamin attributes we inferred using GEMNAST we tested vitamin exchanges’ potential contribution to inferred local networks (IMs). First, we aimed to identify co-abundance/co-occurrence derived IMs^43^ that were composed of five or more members and where at least 50% of members could be matched to one of the strains in our analysis, as specified in Methods. Reliable reports of IMs at the strain level could not be identified, hence we relied on species-level IMs reported by Wang *et al*.^44^ and Zhang *et al*.^45^ which encompass prevalent mice and human gut bacteria, respectively. Strains in our sample were mapped to corresponding species within the reported IMs. Zhang *et al*. sampled the gut microbiome of 19 children with Prader-Willi Syndrome and 21 children with simple obesity at four and two timepoints, respectively, and assessed abundance changes longitudinally (co-abundance). Eighteen prevalent IMs (termed Genome Interaction Groups, GIGs) were identified, four of which met our analysis criteria. Wang *et al*. assessed 101 healthy mice stool samples and identified five IMs (labeled C1 to C5) based on co-occurrence, with three (C1-C3) meeting our analysis criteria (Methods).

Vitamin complementarity networks were built from individual vitamin profiles from strains mapped to an IM and we identified two networks with particular characteristics among the modules that fit our criteria. First, the *Bacteroides* dominated GIG7, for which 13 AGORA strains were mapped, was largely enacted by vitamin prototrophs and three additional strains with a low number of essential vitamins (Table 2). This suggests that IMs that are independent of vitamin exchanges can occur in the gut microbiome.

**Table 2.** Vitamin complementarity network, functional redundancy score and percentage of prototrophs of GIG 7 as reported by Zhang et al. are shown. Original species have been mapped to corresponding AGORA strains, with 13 of the original 15 in the module mapped. Metabolic attributes (green for essential, red for optional) are shown under the corresponding vitamin.

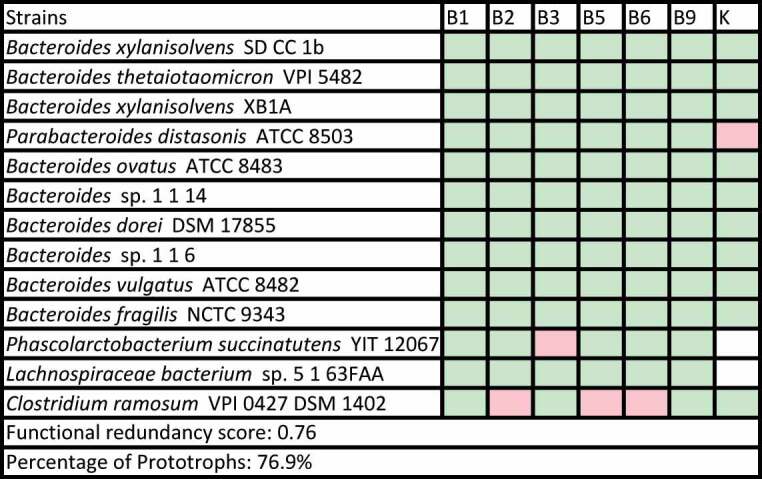

A second IM that presented a distinctive configuration was C2, where strains displayed metabolic complementarity in their vitamin profiles (Table 3), leading us to hypothesize that vitamin interdependencies are driving such a module, where strains act as a codependent entity under vitamin limited contexts. The rest of IMs that fit our assessment criteria displayed vitamin complementarity networks enacted by different proportions of prototrophs and strains with various degrees of auxotrophy. Data pertaining other IMs from Zhang *et al*. and Wang *et al*. can be found in Additional file 5 and Additional file 6, respectively. This analysis shows that a range of configurations in terms of vitamin complementarity can occur in the gut microbiome, ranging from organized vitamin complementarity to relative independence of vitamin-based exchanges.

**Table 3.** Vitamin complementarity network, functional redundancy score and percentage of prototrophs of group C2 as reported by Wang et al. are shown. Original species have been mapped to corresponding AGORA strains, with six of the original eight in the module mapped. Metabolic attributes (green for essential, red for optional and white for non-nutrient) are shown under the corresponding vitamin.

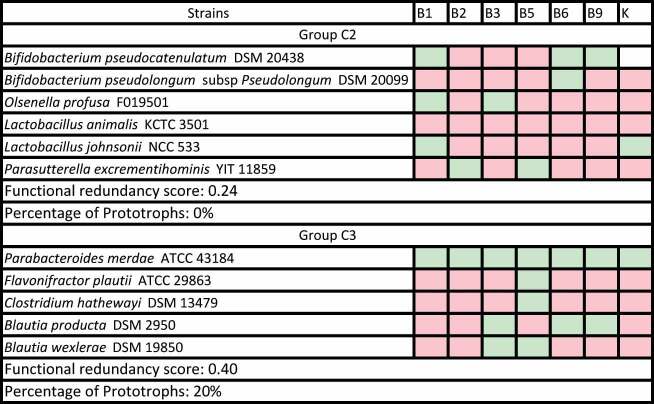

**Figure 4.**
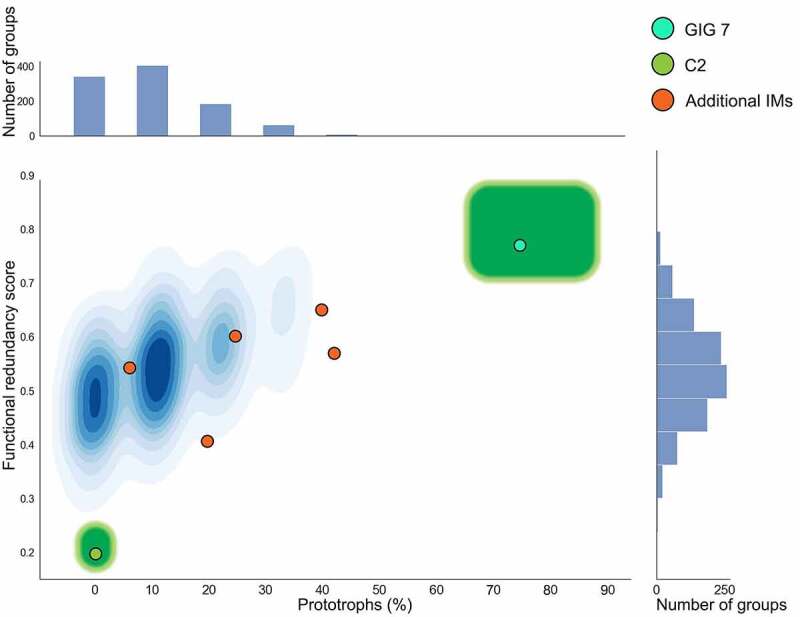
Random pattern of vitamin network configuration based on percentage of prototrophs and average functional redundancy. Randomly generated strain groups clustered at a 0.3 to 0.7 functional redundancy score and 0% to 40% percentage of prototrophs region (blue). Outliers not shown. The observed patterns predicted for analyzed IMs (GIG7: cyan circle, C2: light green circle) fall outside of this range, suggesting the vitamin patterns we propose have a low probability of being a product of randomness. On the other hand, the rest of the assessed IMs (Orange circles) are located remarkedly close to the area where random pattern groups cluster, hinting that such network configurations are less likely to be ecologically meaningful. Following our observations we predict that higher order units driven by vitamin functional attributes would often present characteristics corresponding to the regions highlighted in green surrounding C2 and GIG7. We predict that higher order units with a complementary vitamin profile, such as C2, need to be free of prototrophs and present a very low functional redundancy. Meanwhile, higher order units where vitamin biosynthesis is not a keystone species attribute should present a vitamin profile with a high functional redundancy and a high percentage of prototrophic strains.

To determine the likeliness of the observed complementarity networks being ecologically meaningful or products of randomness, we aimed to assess the random pattern of vitamin network configuration at the IM-level. To do so, we generated 1000 sets of nine randomly selected strains and analyzed the resulting group-level vitamin complementarity networks (Additional file 7). The number of strains in these groups was set to nine as this was the average size of the IMs reported in Zhang *et al*. and Wang *et al*. For our assessment, we focussed on two readily assessable characteristics of the resulting complementarity network: 1) functional redundancy score and 2) percentage of prototrophs. A total of 930 groups presented from zero to two prototrophs, while 958 presented a functional redundancy score between 0.3 and 0.7 (Figure 4). Most IMs that fit our assessment criteria were situated in close proximity to the region where random groups cluster (random pattern) suggesting that the vitamin complementarity networks derived from them are random and that these associations are mainly driven by factors outside of this nutritional dimension. In contrast, four outlier random groups presented a functional redundancy score of less than 0.3 with 0% of prototrophic members, closely resembling C2. However, only one of the four displayed a complete complementary profile (complementarity at every vitamin). Similarly, one outlier random group displayed characteristics similar to GIG7, with a high percentage of prototrophs (55.5%), and a high functional redundancy score (0.86). These results suggest that the probability of GIG7 and C2 vitamin patterns being a result of randomness is close to 0.001%, highlighting that the vitamin profiles we observe in these IMs are ecologically meaningful. Based on these results, we predict that naturally occurring IMs strongly driven by, and independent of vitamin interactions exist by design in the gut microbiome and likely occupy similar regions in the prototroph percentage to functional redundancy plane as C2 and GIG7, respectively (Figure 4). Vitamin exchanges probably still occur within every analyzed IM depending on the nutritional context; however, other aspects of biology are more likely to be driving such groupings. Our analysis strongly suggests that vitamin metabolic attributes influence IM structure in the gut microbiome and provides novel insights of gut ecology in this respect.

## Discussion

Mechanistic drivers of higher order units in the gut microbiome, which are responsible for the biological processes that impact health, can be identified by characterizing the metabolic attributes and nutritional requirements of gut strains. Vitamins participate in crucial intracellular metabolism but also constitute part of the extracellular machinery that contributes to community bioenergetic balance,^11^ positioning them as potential mechanistic drivers of gut higher order units. Relying on GEMNAST, we have performed a metabolic exploration of the vitamin nutritional dimension of gut strain metabolism as represented in AGORA GSMs to determine the role of this aspect of strain metabolism in community structure. By applying our framework, we identify essential (must be provided by the environment) and optional (can be synthesized when not available) vitamins, and non-nutrients for 816 gut microbiome strains. Eleven vitamin capability groups that spread along an auxotrophy to prototrophy spectrum and exceed phylogenetic relatedness are then derived from such vitamin metabolic attributes. Strains often present complementary vitamin attributes, which suggests that vitamins can be exchanged between human gut commensals.^18,46,47^ Based on this premise, we test vitamin exchange potential as one of the explanatory elements promoting the emergence and permanence of higher order units within the gut microbiome. Overall, our analysis suggests that vitamin metabolic attributes play a role shaping both modular and broad community structure.

Assessment of vitamin metabolic attributes of gut strains allowed us to test vitamin exchanges as potential drivers of local networks. We identify two IM configurations that are fundamentally different than the observed random configuration pattern. C2 is characterized by vitamin complementarity profiles across all members and is hypothesized to exist as a codependent entity under vitamin scarcity. GIG 7, dominated by *Bacteroides*, represents an IM largely independent of vitamin exchanges. *Bacteroides* are characterized by their wide saccharolytic potential,^9^ making GIG7 a potentially longitudinally stable module. It is likely that such modules are prevalent in *Bacteroides* enterotypes, which would partially explain their resilience to dietary interventions.^41,48^ We also identify marked differences between enterotype indicators’ vitamin metabolic attributes when mapped to vitamin capability groups. Our findings suggest that limited environment vitamin availability importantly impacts *Prevotella* strains, unless paired with select strains with complementary vitamin biosynthesis attributes, while *Bacteroides* display independence in these terms.

*Bacteroides* enterotypes have been associated with long-term high fat/high protein diets,^49^ which tend to be low in the majority of vitamins we test here.^50^ Hence, low vitamin environmental availability could favor *Bacteroides* dispersal and contribute to the establishment of a *Bacteroides* enterotype. The *Prevotella* enterotype has been associated with agrarian diets and veganism,^49,51,52^ which are often also correlated with strains from genera/families we identified to have complementary vitamin functional profiles such as *Ruminococcaceae, Lactobacillaceae*.^51,53^ Hence, we speculate that agrarian diets may contribute to the emergence and permanence of the *Prevotella* enterotype, where IMs, enacted by *Prevotella* and other fiber degrading strains, are supported by complementary vitamin metabolic attributes, among other factors.

In our analysis, we find that 43 to 74% of strains in our sample can synthesize one of the assessed vitamins. A previous analysis based on genome annotation reported similar outcomes (40–65%).^20^ Although similar, our analysis also allowed us to identify vitamins that are non-nutrients for a portion of strains. Furthermore, thanks to the granular level of control GSMs offer over culture media, we can confidently assert that tested strains had no access to any of the vitamins, or analogues, when these were being examined, complicated to guarantee during *in vitro* assessments. Importantly, however, our results are highly dependent on the nutritional contexts generated from our UDM and alterations to such will change the observed outcomes. Nevertheless, GEMNAST can be applied to explore new nutritional contexts as GSMs readily allow modeling of diets with detailed nutrient composition.

According to our model-based findings, vitamins are not actively exported to the extracellular space. If true, this leads us to hypothesize that mechanisms of vitamin exchanges may occur passively, for example, following cell lysis.^13^ We note that undescribed active mechanisms mediating vitamin sharing would not be captured by AGORA. For example, *Faecalibacterium sp*. KLE1255 failed to grow under commercially available quinones but did grow when cocultured with *Escherichia coli*, leading to the hypothesis that the later can deliver the extremely hydrophobic quinones to the former through membrane vesicles.^28^ Released vitamins are likely to be in their respective active forms, which might not be usable by every strain unless they present specialized transport mechanisms. For example, we find that the most actively synthesized form of B1 is thiamine pyrophosphate (TPP), which cannot freely diffuse through the cell membrane. However, there is evidence that some bacteria can uptake TPP through specific transmembrane channels.^54^ We also identify that B12 (cobalamin) is an optional vitamin for a large proportion of strains, while essential for none. Our analysis includes three B12 analogues, however, eight corrinoids (B12-like molecules) have been identified in the human gut^46^ and *Bacteroides thetaiotaomicron* transporters are compatible with such compounds.^19^ This suggests that corrinoids might serve as exchange currencies in the gut. AGORA does not capture this aspect of the dimension which might explain the high number of cobalamin-independent strains. Moreover, studies have shown that cobalamin requirements can be replaced with the addition of methionine to the media,^55^ an important component of UDM.

Despite its many practicalities, GSMs-based assessment carry certain caveats. Requiring convergence from other fields of research, GSMs suffer of similar limitations such approaches face, resulting in potential inaccuracies even after thorough curation. For example, GSMs rely on upper rate bounds to model cellular metabolism which are mostly known only for model organisms.^56^ In addition, *in vitro* experimentation is required to ascertain such rates, which will not only help increase GSM accuracy but will allow them to capture the biological nuances we observe around growth rate proxies, such as variation in OD. In addition, raw AGORA models face limitations when performing quantitative predictions without prior manual curation due to a tendency of some of the models to predict inflated growth rates.^57^ As our understanding of the biology of gut microbes increases, along with the development of more efficient computational tools, the time-demanding model curation of the 816 AGORA strains will become more feasible. Nevertheless, the current models are an invaluable resource to perform the extremely high-throughput analysis we describe here. We rely on a conservative minimum growth rate threshold of 0.09/h, as failure to surpass such a threshold strongly implies that a given strain does not have adequate metabolic means to sustain growth in the explored nutritional environment, conveying a high specificity to our results.^55^ Finally, current GSMs struggle to represent other relevant aspects of gut biology such as host-derived pressures and nutrient heterogeneous distribution. Such factors need to be addressed to more accurately recapitulate inter-strain interactions in the gut. Future efforts should account for such nuances when exploring vitamin metabolism.

The body of work we present here constitutes an important step toward understanding the emergent dynamics and structures within the gut microbiome. Analysis of the metabolic attributes of individual gut strains in an underexplored nutritional dimension allowed us to propose plausible mechanistic explanations that support enterotype identity and the observed co-abundance profiles within the gut, offering a novel perspective into the gut ecosystem. Further, the database we have generated can be used to inform future efforts including the design of minimal media for specific strains, which will in turn allow us to curate and assemble more accurate GSMs. Finally, GEMNAST can be employed to survey other relevant nutritional dimensions and provide a more comprehensive understanding about the survival strategies that explain the origin of the community-level outputs that modulate health.

## Materials and methods

### Computational resources

Genome scale models used in this study are part of AGORA (Automatically generated genome-scale metabolic reconstructions)^29^ which in total is composed by 818 strains. Currently, two versions of AGORA are available for download from the Virtual Metabolic Human database;^58^ here we employ the *without mucins* version. We included 816 of the available AGORA strains in our analysis with two excluded due to inconsistencies as describe below. Metabolite flux for each GSM was modeled using the constraint-based reconstruction and analysis (COBRA) toolbox for the Python coding language (cobra.py).^36^ Metabolite flux analysis was performed using cobra.py and Flux Balance Analysis (FBA). FBA calculates growth in terms of biomass generation in grams of dry weight per hour (gDW/h) based on nutrient fluxes. Cobra.py further allows a) inspection of individual metabolic reactions and metabolite consumption and generation and b) seamless addition or removal of specific molecules to growth media.

### Universally defined media (UDM) design and experimental design

**Figure 5.**
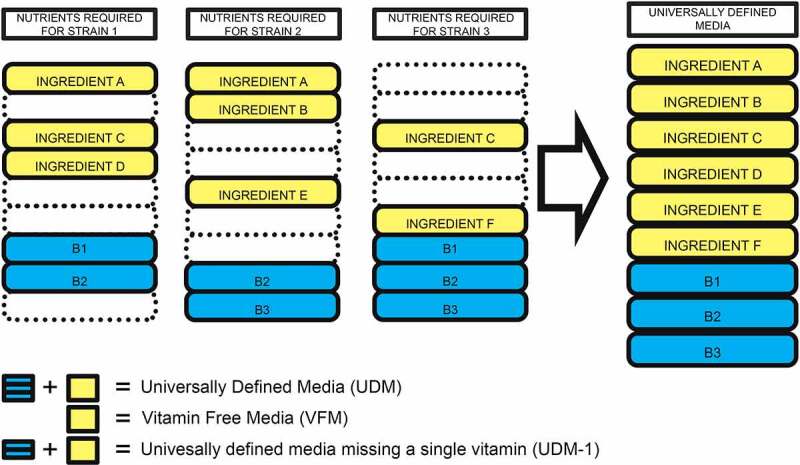
Simplified Universally Defined Media (UDM) design: Sets of nutrients required for three individual hypothetical AGORA strains to achieve optimal growth are combined to generate a hypothetical, simplified version of the UDM we utilized for our study. Yellow boxes exemplify UDM ingredients other than vitamins while blue boxes exemplify whole vitamin families. Vitamin-free UDM (VFM), is used as base media to infer strain essential vitamins. UDM-1 (a group of eight different media) were also derived from UDM by removing a single vitamin family shown in Table 1 from UDM. UMD-1 were employed to identify optional vitamins.

Universally defined media (UDM) was designed by generating anaerobic minimal media for every strain in AGORA. The minimal_medium method in the cobra.py library was employed to generate media with the basic set of nutrients required for each strain to achieve a growth rate of 0.8 h¯^1^ (value suggested in the minimal_medium documentation), a proxy for optimal growth rate. A determined set of nutrients for strain *Lactobacillus helveticus* DPC 4571 could not be generated which led to the exclusion of this strain from further analyses. The resulting set of nutrients for each strain was broken into its constituents and these were compiled into anaerobic UDM (Figure 5). Nutrients were categorized into 11 groups: simple sugars, amino acids, dipeptides, fatty acids, bile acids, cations, anions, metals, main vitamins (those explored in this study), secondary vitamins and other. A detailed list of the components in our UDM can be found in Additional file 8. The resulting anaerobic UDM was used as a base for our growth experiments. An initial growth test using UDM was performed where we confirmed that every strain was capable of growing in it.

### GSM structure and vitamin selection

GSMs are mathematic reconstructions of the metabolic reactions encoded in an organism’s genome, including reversible and irreversible exchange/transport reactions. GSMs allow for the modeling of two virtual compartments: an extracellular one and an intracellular compartment. During metabolic modeling, strains can interact (uptake, secrete) with metabolites in the media if it presents the corresponding exchange/transport ‘reactions’. Similarly, if a GSM contains reactions that lead to the synthesis of a given metabolite, it will be capable of synthesizing it under the right media. Importantly, GSMs can be constrained to consume, synthesize, or export a specific metabolite if they have the appropriate reactions.

Exploration of reactions within AGORA strains identified vitamins from the vitamin B family and Quinones (Menaquinones and Ubiquinone) and their analogues (Table 1) taking part of such metabolic networks; other vitamins (A, C, D, and E) as well as other quinones were not identified and were not considered for our analysis. A preliminary analysis showed that biotin (B7) did not play a meaningful role among AGORA strains, which we considered to be a biological inconsistency, and consequently it was excluded from our current analysis.

### Determining essential and optional vitamins

To infer essential nutrients of strains, we developed a Python pipeline that models strain growth in defined media and compiles outcomes in a single Boolean table. The script requires three inputs: a) a directory where to read strain names and GSMs from; b) a list of media ingredients to serve as base media; and c) a set of nutrients on which to determine nutritional requirements which should not be part of the base media. A minimum growth rate threshold can also be determined within the script. Nutritional environments are designed based on the number of explored nutrients (input c) following a combinatorial design where nutrients are introduced individually or in combination to comprehensively explore every possible combinatorial scenario. Starting with base media, a first strain is cultured, its growth rate is assessed, and an outcome is recorded. The cycle continues by adding first individual nutrients (input b) and then increasingly complex combinations of them to base media, which are removed after growth rate assessment, before starting a new round (Figure 1a). Strains are explored one by one, one nutritional environment at a time and growth rate (gDW/h, biomass generation) is calculated in every culture. The main output from this analysis is a comma separated values (.csv) file that contains a Boolean table with strains as rows and nutritional environments as columns. If a strain’s growth rate is higher than the defined threshold a positive outcome is recorded in the Boolean table,^1^ otherwise a negative outcome (0) is recorded. Our incremental approach (from zero to all the explored nutrients) expedites the identification of the minimal requirements a strain has in terms of the explored set of nutrients; unless a strain is capable of growing under a variety of explored conditions the first instance where a strain presents meaningful growth is the one that introduced the set of required nutrients to the media. To explore extrinsic vitamin requirements we removed the vitamins of interest from our original UDM obtaining vitamin-free UDM (VFM, Figure 5) which was used as base media (input b). Since our UDM was purposefully designed to be a rich medium, removal of the explored vitamins ensured these were the only limiting nutrients. *Clostridium sporogenes* ATCC 15579 displayed an incompatibility with the employed solvers and irregular results, leading us to exclude it from our study. A total of 816 AGORA strains were assessed for their essential vitamins (input a). A threshold of 0.09 gDWh¯^1^ growth rate (six to eight hours doubling time) was established on the basis that any strain growing at a lower rate would present relatively low probabilities of surviving in the human gut, where transit time can be shorter than 14 hours in some individuals.^59^ Therefore, this threshold represents a theoretical minimum growth rate for gut survival as less than two replication cycles would not guarantee permanence in the colon. The vitamin families specified in Table 1 were selected for our combinatorial analysis (input c) in order to determine which combination of vitamins a particular strain needed for growth (Figure 1d, “Require”).

**Figure 6.**
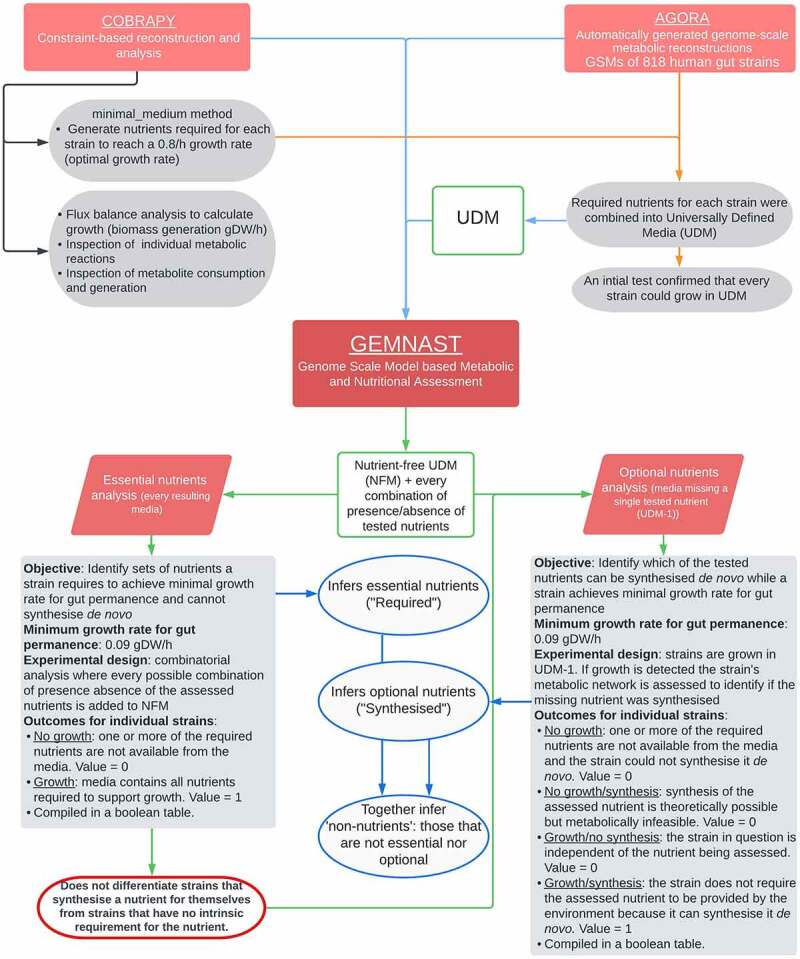
GEMNAST flowchart and its integration with AGORA and cobra.py. AGORA GSMs and cobra.py libraries and methods were employed in the design of UDM and GEMNAST. GEMNAST is composed of two branches that complement each other: nutritional requirements and biosynthetic capabilities analyses. GEMNAST’s nutritional requirements analysis assesses growth vs no growth and performs a combinatorial analysis. GEMNAST’s biosynthetic capabilities analysis assesses growth and synthesis and does not requires a combinatorial analysis. Base media for each analysis are fundamentally different. Each branch compiles outcomes in individual Boolean tables.

A second pipeline was developed to infer optional nutrients of strains, given that our essential nutrient analysis does not differentiate strains that biosynthesise nutrients for themselves from those that simply do not require the nutrient for growth. Similar to our essential nutrients analysis, this script requires three inputs: a) a directory where to read strain names and GSMs from; b) a list of media ingredients to serve as base media; and c) a set of nutrients of interest which *should* be part of the specified base media. This assessment also analyses one strain at a time but does not perform a combinatorial analysis. Instead every time synthesis of a metabolite is assessed, the nutrient and its analogues are removed from base media (UDM – 1). Following nutrient removal, a strain is cultured in the resulting media. The pipeline assesses two aspects of strain metabolism: its capability to synthesize the nutrient in question while growing above a previously specified threshold. To identify if a metabolite was synthesized, the metabolic network is assessed in search for reactions that led to synthesis and utilization of the nutrient of interest. Outcomes are registered in a Boolean table with strains as rows and resulting nutritional environments as columns where cultures in which strains grew above the determined threshold and synthesized the nutrient in question are assigned a value of one^1^ (Figure 1d, “Synthesises”). If the reported growth rate is below the given threshold and/or if the strain did not synthesize the assessed compound a zero (0) is assigned to that culture. To infer optional vitamins, we utilized our UDM as a base which included all the explored vitamins (input b) and the same growth threshold specified above (0.09 gDWh¯^1^). The vitamin families specified in Table 1 were selected for this analysis in order to determine which were synthesized by a particular strain (input c). Eight hundred and sixteen AGORA strains were assessed (input a). There was a small number of cases where a strain presented ‘non-nutrient’ vitamins, categorized as “None” in Figure 1, panel D. A modified version of our second pipeline was employed to assess vitamin export capabilities with the only differences that 1) growth and secretion would be assessed instead of growth and synthesis and 2) strains were constrained to export the vitamin assessed to cover for contexts where secretion was optional. Together, this method to explore GSM nutritional requirements, biosynthetic capabilities, and metabolite export is termed GEMNAST (Genome scale model Metabolic and Nutritional AssessmenT). Scripts can be accessed online: https://github.com/jmol0917/GEMNAST_pub.git. A flowchart summarizing the main aspects of GEMNAST, AGORA, cobra.py and the methods involving UDM, VFM, and UDM-1 is provided in Figure 6.

### *Determining vitamin requirement* in vitro

Soto-Martin *et al*. determined maximum optical density (maxOD) by culturing strains in the study in media with all the explored vitamins. Strains were later cultured in media with a single missing vitamin and OD was compared against maxOD. To validate our results, we considered that a strain that achieved over 80% maxOD (optimal growth) did not require the missing vitamin to be externally sourced, while anything below 20% was interpreted as it requiring the vitamin from an external source; values between 20% and 80% maxOD were considered as inconclusive (Additional file 1).

### Clustering and kernel density estimation (KDE)

Strains were clustered based on individual growth profiles using the clustermap method from the Python data visualization library Seaborn^60^ which generates a matrix dataset and a hierarchically clustered heatmap. The two data visualization tools, t-distributed stochastic neighbor embedding from the TSNE scikit-learn Python module,^61^ and kernel density estimation, form the SciPy library for Python,^62^ were used for cluster and cluster density visualization on a two-dimensional plane (Additional file 4). Resulting matrixes contributed to the vitamin capability groups curation process.

### Phylogenetic tree assembly

Taxonomy IDs of 806 strains in our dataset were retrieved from the Virtual Metabolic Human database^58^ and were used to assemble a .phy file using NCBI’s online taxonomy browser.^40^ The interactive Tree Of Life online tool^63^ was accessed to create a graphic representation of the resulting phylogenetic tree and strain names were highlighted based on the vitamin capability group they were mapped. Strains that were not mapped to any group are not highlighted.

### Vitamin interaction module assessment

Interaction module tables were generated from IMs from Wang *et al*.^44^ and Zhang *et al*.^45^ that 1) were composed of five members or more and 2) at least 50% of members could be matched with one of the strains in our analysis. If multiple strains could be mapped to the same species multiple versions of the same IM were generated, one with each of the potential candidates and the network configuration of each version was assessed. The strain-level IMs presented in this study either showed minimal inter-version variation or only a single version could be generated from its original IM.

A Python script was developed to generate random groups of strains together with their vitamin requirements and biosynthetic capabilities using the pandas data analysis and manipulation tool.^64^ Scripts employed to generate interaction tables and randomly generated groups can be accessed online: https://github.com/jmol0917/fcmmtGSM.git. The jointplot seaborn class^60^ was used to generate a bivariate plot (Figure 4) of the 1000 random groups. To obtain the average functional redundancy of the randomly generated group and IMs, we employed the following formula:

$$\frac{vs/nv}{nm}$$

Where *vs* corresponds to total number of vitamins synthesized per group/IM; *nv* is the number of assessed vitamins, in this case seven; and *nm* corresponds to the number of members in a group/IM.

## Supplementary Material

Supplemental Material

## Data Availability

All data generated or analyzed during this study are included in this published article and its supplementary information files.
