# Supplementary material for: High throughput genome scale modeling predicts microbial vitamin requirements contribute to gut microbiome community structure
Source: Gut Microbes. 2022 Sep 8;14(1):2118831. doi: 10.1080/19490976.2022.2118831 (PMC9480837; doi:10.1080/19490976.2022.2118831)
Supplement: Supplemental Material [file KGMI_A_2118831_SM6552.zip › Additional file 1.pdf]

Strain	B1	B2	B3	B5	B6	B9	B12	Source
Faecalibacterium prausnitzii A2-165								Soto-Martin *et al.* (*in vitro*)
								Present study (*in silico*)
Faecalibacterium prausnitzii SL3/3								Soto-Martin *et al.* (*in vitro*)
								Present study (*in silico*)
Subdoligranulum variabile DSM 15176								Soto-Martin *et al.* (*in vitro*)
								Present study (*in silico*)
Eubacterium hallii DSM 3353								Soto-Martin *et al.* (*in vitro*)
								Present study (*in silico*)
Coprococcus catus GD/7								Soto-Martin *et al.* (*in vitro*)
								Present study (*in silico*)
Clostridium sp. L2-50								Soto-Martin *et al.* (*in vitro*)
								Present study (*in silico*)
Eubacterium rectale M104/1								Soto-Martin *et al.* (*in vitro*)
								Present study (*in silico*)
Roseburia intestinalis L1-82								Soto-Martin *et al.* (*in vitro*)
								Present study (*in silico*)
