# Supplementary figures and images for: High throughput genome scale modeling predicts microbial vitamin requirements contribute to gut microbiome community structure
Source: Gut Microbes. 2022 Sep 8;14(1):2118831. doi: 10.1080/19490976.2022.2118831 (PMC9480837; doi:10.1080/19490976.2022.2118831)

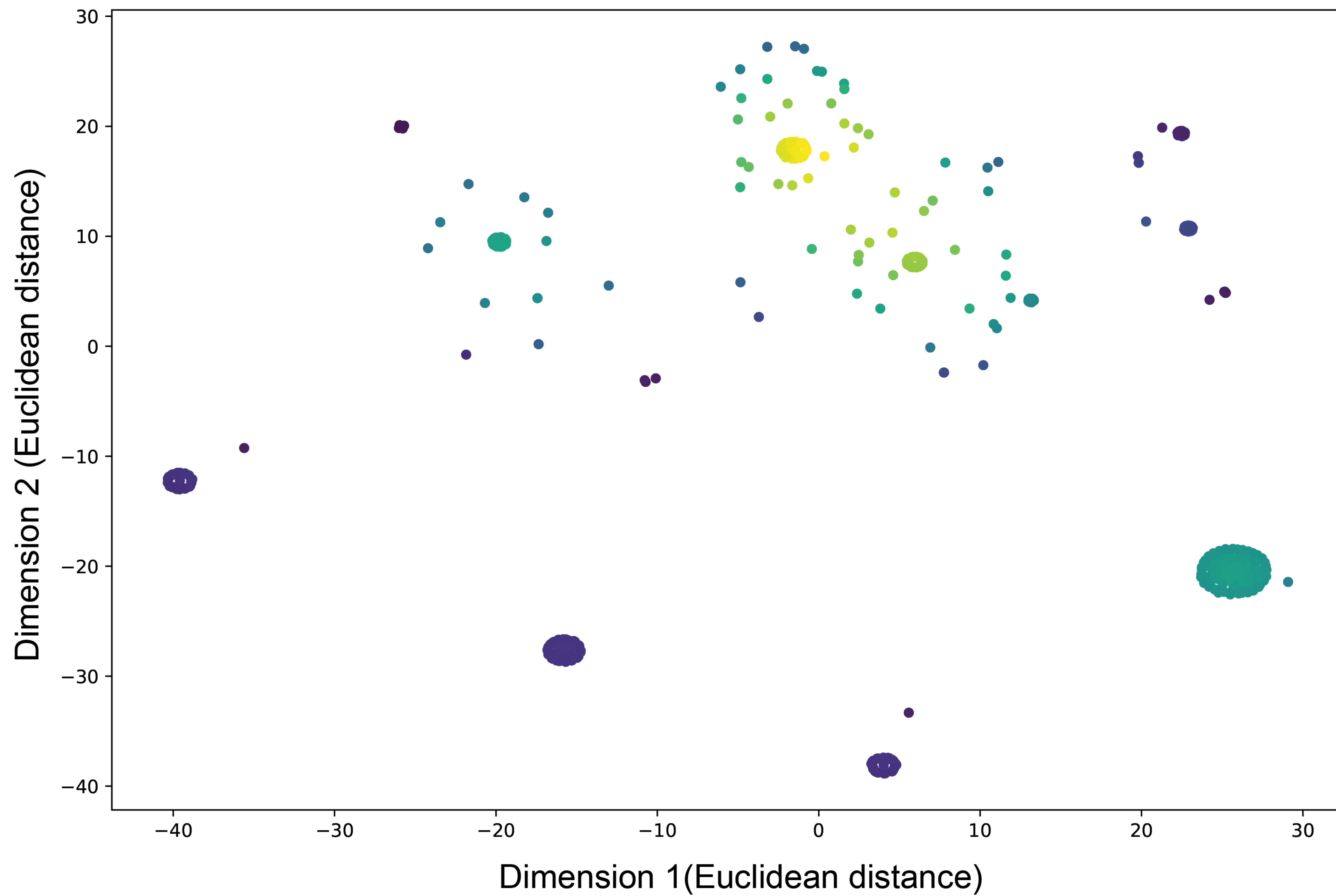

Supplement: Supplemental Material [file KGMI_A_2118831_SM6552.zip › Additional file 4.pdf]
